# Excessive Dynamic Airway Collapse After Entecavir Use in a Patient With Pegylated Interferon-Induced Undifferentiated Connective Tissue Disease and Entecavir Use to Prevent Hepatitis B Virus Reactivation Upon Giving Rituximab

**DOI:** 10.7759/cureus.62835

**Published:** 2024-06-21

**Authors:** Muhammad Zain Akhtar, Ghias Un Nabi Tayab, Muhammad Imran Nawaz, Iqra Seher

**Affiliations:** 1 Department of Medicine, Mayo Hospital, Lahore, PAK; 2 Department of Medicine, Royal Preston Hospital, Preston, GBR; 3 Department of Gastroenterology and Hepatology, Lahore General Hospital, Lahore, PAK; 4 Department of Medicine, Lahore General Hospital, Lahore, PAK

**Keywords:** delayed complications, hepatitis c virus treatment, entecavir, undifferentiated connective tissue disease, pegylated interferon

## Abstract

Pegylated interferon-alpha (PEG-IFN-α) is an antiviral medication used to treat chronic hepatitis C virus (HCV) and hepatitis B virus (HBV) infections. It may result in rare but severe side effects, such as undifferentiated connective tissue disease (UCTD) and excessive dynamic airway collapse (EDAC), which can occur as delayed complications of PEG-IFN-α-induced UCTD. In cases where these complications arise, entecavir, employed for treating HBV infection, may be considered. A 49-year-old female patient, monitored for nine years with HCV and a viral load of 1.5 million, genotype 3, and normal liver function tests (LFTs), possibly acquired the infection from her HCV-positive husband. The patient was initially treated with PEG-IFN-α (IFN-α-2b, 100 µg/week subcutaneously) and ribavirin (RBV, 500 mg/twice daily). Following the sixth injection, the patient exhibited symptoms, including shortness of breath and cough, leading to limited daily activities. Subsequent high-resolution computed tomography (HRCT) showed interstitial pneumonitis (IP) signs. She was given a high dose of steroids. Over the next two to four weeks, the patient experienced Raynaud's phenomenon, skin tightening, joint pains, and dryness of the eyes and mouth. The antinuclear antibody (ANA) test was negative, while the extractable nuclear antigen (ENA) test showed equivocal anti-Smith antibodies (6.38). Rheumatoid factor (RA) factors were mildly positive, and pulmonary function tests (PFTs) indicated a restrictive pattern. The patient was intolerant to hydroxychloroquine (HCQ) and azathioprine (Imuran) 500 mg, subsequently receiving mycophenolate mofetil 500 mg/thrice daily. Despite four years of treatment, UCTD due to PEG-IFN-α remained difficult to control; however, IP responded well to steroids. Rituximab pulse therapy was planned before the initiation; serological tests showed positive anti-HBs with a titer of 17.02, positive anti-HBc, but negative HBsAg and undetectable HBV viral load, indicating immunity to HBV due to natural infection. Given the potential for rituximab to cause immunosuppression and HBV reactivation, entecavir treatment was started and continued for 18 months. The patient was followed for another five years, during which her LFTs and viral markers showed stability. However, after nine years of PEG-IFN-α-induced UCTD disorder, she experienced a reoccurring cough but was unresponsive to steroids that were against her suspicion of a flare of IP. A subsequent dynamic CT scan detected a 75% trachea collapse while in a supine position, indicating a potential complication termed EDAC. This EDAC could not be linked to PEG-IFN-α-induced UCTD disorder or EDAC after the use of entecavir in a patient with PEG-IFN-α-induced UCTD disorder. Treatment of such complex patients requires flexible, specific treatment plans and continuous monitoring. This case emphasizes the need for caution in patients with a history of IFN-induced disease and the possibility of late effects and possible effects of the use of entecavir in a patient with PEG-IFN-α-induced UCTD. To the best of our knowledge, this is the first case reported as EDAC, a possible delayed complication of PEG-IFN-α plus ribavirin or entecavir in a patient with PEG-IFN-α-induced UCTD.

## Introduction

Pegylated interferon-alpha (PEG-IFN-α) is a commonly used antiviral drug for treating chronic hepatitis C virus (HCV) infection. However, it can have rare but severe side effects, such as undifferentiated connective tissue disease (UCTD), a condition characterized by symptoms suggestive of an autoimmune disease without meeting specific criteria for a specific connective tissue disease [1]. Another significant complication is interstitial pneumonitis (IP), which involves inflammation and fibrosis of the lung tissue, leading to breathing difficulties and impaired lung function [2]. Excessive dynamic airway collapse (EDAC), the collapse of the airway during exhalation, is another severe, albeit rare, complication. HCV is a major global health concern, and treating it effectively is crucial to preventing severe liver disease. PEG-IFN-α, especially when combined with ribavirin, has been a cornerstone in HCV treatment, although newer therapies have emerged. Entecavir, typically employed for treating hepatitis B virus (HBV) infection, may be considered, especially in patients with concurrent HCV and HBV co-infections. It is essential for patients who are at risk of HBV reactivation, such as those receiving immunosuppressive therapies like rituximab. Pulmonary complications may be rare but are associated with the use of IFN and entecavir in the treatment of HCV. These pulmonary problems are rare and occur with an overall incidence of less than 1% [3]. The spectrum of these complications is notable and includes conditions such as pulmonary sarcoidosis, IP, bronchiolitis obliterans, organizing pneumonia, pleural effusion, exacerbation of bronchial asthma, acute respiratory distress syndrome (ARDS), and EDAC [4].

EDAC is caused by the invagination of the posterior wall of the trachea during exhalation. This condition often occurs as a secondary effect of the dynamic forces associated with hyperinflation in obstructive lung disease [5-7]. Both diseases can severely impair the quality of life of patients undergoing therapy with PEG-IFN-α [8]. For many years, IFN-α was the only effective treatment for chronic HCV [9]. However, Karim et al. [10] have shown that combination therapy of IFN-α and ribavirin (RBV) leads to a more sustained response than IFN-α alone. The combination of ribavirin with PEG-IFN-α showed improved efficacy, superior to PEG-IFN-α alone, leading to an approximate 30% increase in sustained virological response (SVR) rates [11]. This case study highlights a 49-year-old female patient who developed multiple severe complications, including UCTD and IP, after being treated with PEG-IFN-α and ribavirin for HCV. The patient later received entecavir to prevent HBV reactivation during rituximab treatment. Uniquely, nine years post-treatment, she developed EDAC, a rare complication that could potentially be linked to her previous treatments. This report underscores the complexity of managing such patients and the need for long-term monitoring and individualized treatment plans. To the best of our knowledge, this is the first reported case of EDAC as a possible delayed complication of PEG-IFN-α plus ribavirin or entecavir.

## Case presentation

**Figure 1:**
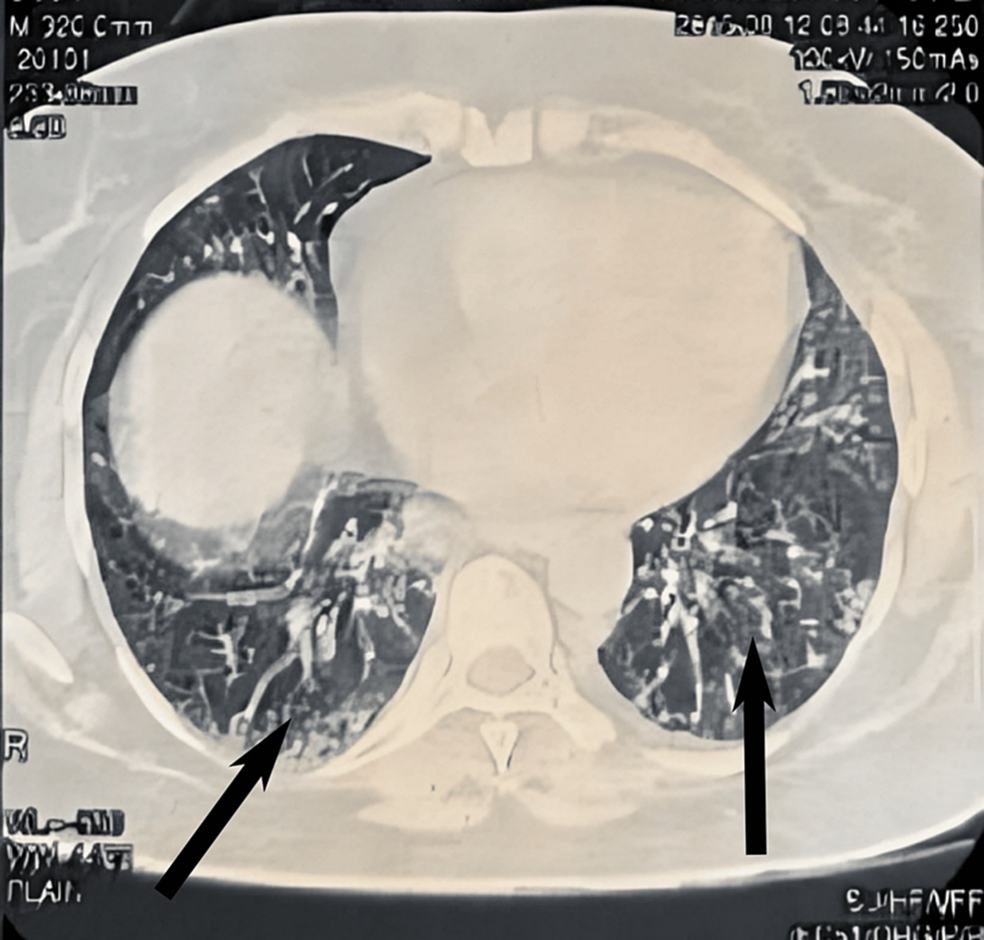
A cross-sectional view of a chest computed tomography (CT) scan image shows interstitial pneumonitis (IP) following treatment with pegylated interferon-alpha (PEG-IFN-α). Arrows indicate the presence of IP after PEG-IFN-α. HRCT reveals a ground glass appearance in the left lower lobe and a reticular pattern in the right lower lobe.

**Figure 2:**
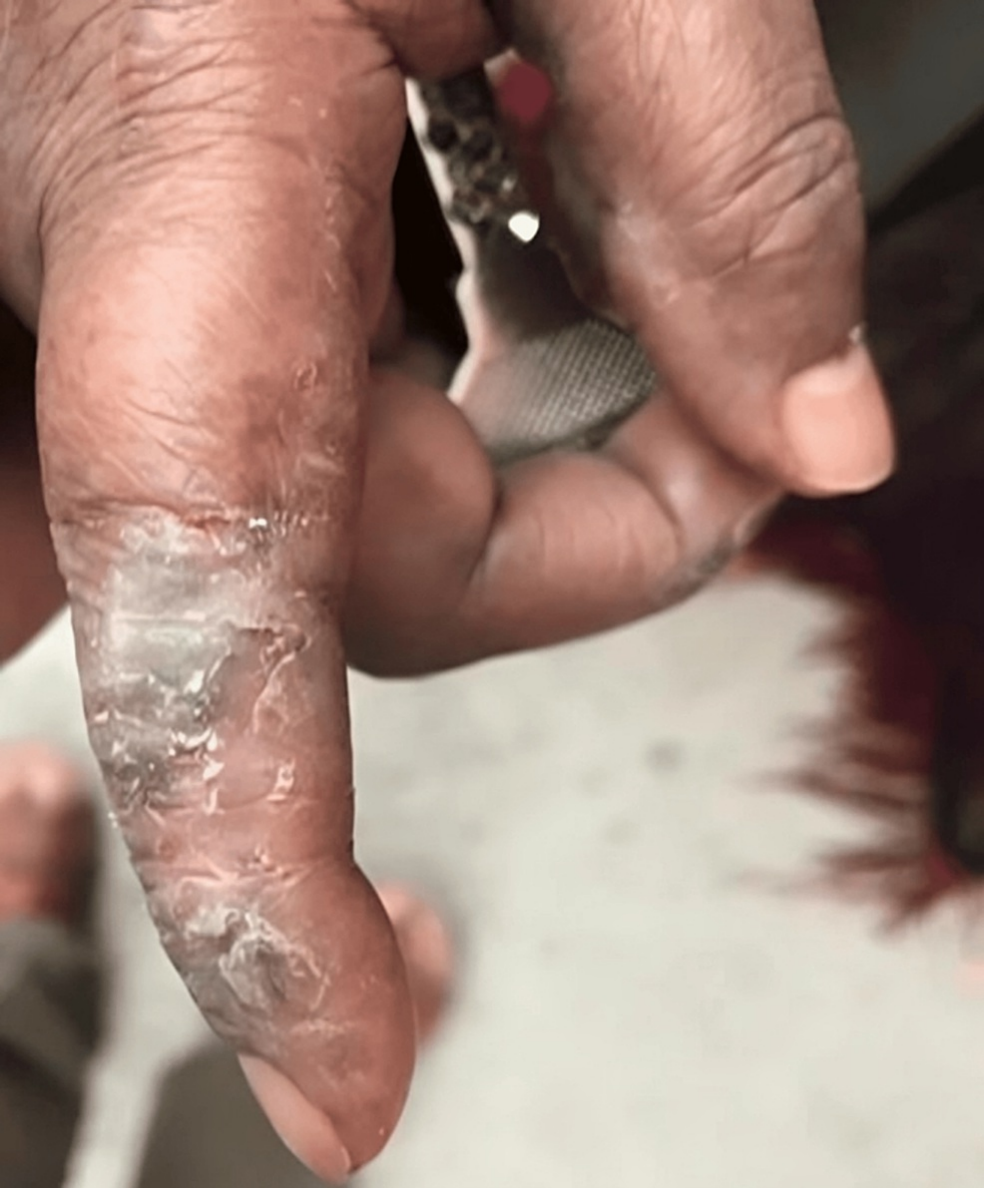
Patient's finger image reveals undifferentiated connective tissue disease (UCTD) manifestations. The patient exhibits a blue discoloration of the finger accompanied by eczematous changes.

A 49-year-old female with a history of chronic HCV infection was treated with PEG-IFN-α (IFN-α-2b 100 µg/week subcutaneously) and ribavirin (500 mg/twice a day). The patient initially presented with a viral load (1.5 million, genotype 3) but normal liver function test (LFTs). After the sixth injection of PEG-IFN-α, she developed shortness of breath and a persistent cough, significantly limiting her daily activities. High-resolution computed tomography (HRCT) revealed signs of IP (Figure 1); subsequently, she experienced indications of UCTD, including Raynaud's phenomenon, discoloration of fingers (Figure 2), joint pains, skin tightening, and dryness of the eyes and mouth. Her antinuclear antibody (ANA) test was negative, but the extractable nuclear antigen (ENA) test showed equivocal anti-Smith antibody levels (6.38), and rheumatoid factor (RA) was mildly positive. PFTs indicated a restrictive pattern. She began treatment with hydroxychloroquine (HCQ) but experienced side effects, including gastrointestinal discomfort (nausea and diarrhea) and headaches, and was intolerant of azathioprine (Imuran) due to nausea, fever, and fatigue, after which she was given mycophenolate mofetil, 500 mg three times a day. Despite four years of treatment, UCTD remained challenging, but IP responded well to steroids. Rituximab was planned for further treatment, and pre-therapy serological tests showed positive anti-HBs with a titer of 17.02 and anti-HBc but negative HBsAg and undetectable HBV viral load, indicating immunity to HBV. Entecavir was administered for 18 months to prevent HBV reactivation during rituximab therapy.

**Figure 3:**
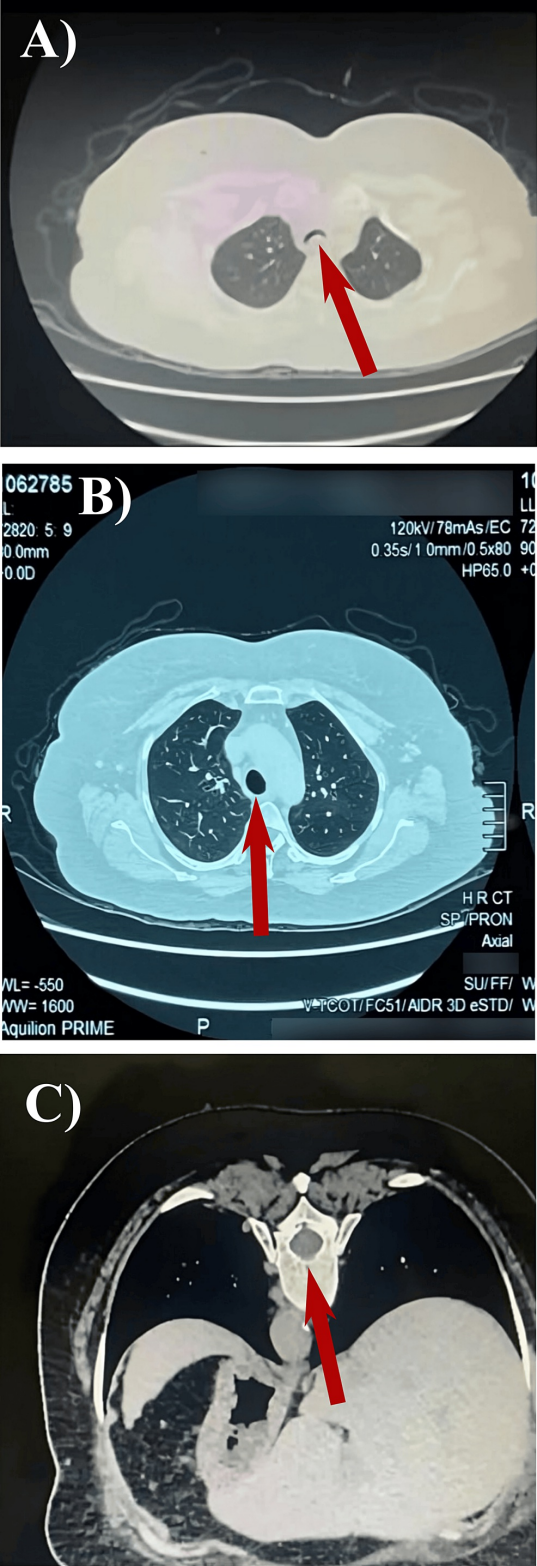
A dynamic high-resolution computer tomography (HRCT) reveals patent airways during inspiration but shows a 70% reduction in cross-sectional area during expiration and inspiration in the supine position (A). HRCT scan of normal trachea (B). Additionally, the trachea remains patent in the prone position, indicated by an arrow (C). The collapse of the trachea was causing severe dry cough and episodes of dizziness, both symptoms consistent with EDAC.

Over five years of follow-up, her LFTs and viral markers remained stable. Nine years after PEG-IFN-α treatment, she presented with a recurrent cough unresponsive to steroids, ruling out an IP flare. A dynamic CT scan revealed a 75% collapse of the trachea in the supine position (Figure 3), diagnosing EDAC. This case suggests EDAC as a potential delayed complication of PEG-IFN-α and ribavirin or entecavir in patients with PEG-IFN-α-induced UCTD.

## Discussion

The administration of PEG-INF-α therapy, both alone and in combination with ribavirin or PEG-IFN-α/ribavirin, has been linked to the onset and worsening of autoimmune diseases, cardiotoxic effects, and, in some cases, sudden death [12]. Chin et al. [13] reported a case involving a female patient who needed periodic prednisolone pulse therapy for symptom management. Abi-Nassif et al. [14] reported that a male patient suffered from severe IP and ultimately passed away due to acute respiratory distress syndrome (ARDS) and septic multiorgan failure. Another case report revealed IP associated with PEG-IFN in a 68-year-old male patient with chronic HCV infection. Thirty-six weeks after starting therapy with PEG-INF-α and ribavirin, this problem became apparent. The patient's condition did not improve even after discontinuing treatment with PEG-IFN/ribavirin until he began steroid therapy [15]. The toxicity associated with IFN is generally linked to the dosage and length of treatment, with higher doses and longer durations typically leading to increased toxicity. However, a definitive correlation between the dosage and the probability of side effects has not been established. PEG-IFN-α results in elevated IFN levels that are similar to those of conventional IFN-α; there is a tendency for pulmonary toxicity to occur more often with the long-acting PEG-IFN-α therapy [16]. The involvement of ribavirin in causing pulmonary side effects when used with PEG-IFN-α is still a matter of conjecture.

Given that dyspnea and cough are recognized adverse effects of ribavirin, it raises the possibility that ribavirin might independently contribute to pulmonary toxicity. Nonetheless, since cough and dyspnea are frequently occurring side effects, they could potentially obscure the identification of lung diseases related explicitly to IFN treatment [17]. Entecavir serves as a preventive strategy to inhibit the reactivation of HBV in a patient with a previous HBV infection who is receiving rituximab, an immunosuppressive therapy. As an antiviral effective in controlling HBV, entecavir suppresses HBV DNA replication, thus minimizing the risk of potentially severe and life-threatening HBV reactivation during the immune-compromised state induced by rituximab treatment [18].

The pathogenesis of PEG-IFN-induced IP is not fully understood. Still, it is thought to stem either from the drug's direct toxic effect on lung tissue or from its impact on immune pathways [19]. While most cases of IFN-related lung toxicity in chronic HCV patients can be reversed by discontinuation of the treatment, there are only four known cases where IP continued even after stopping PEG-INF-α/ribavirin and starting immunosuppressive therapy.

## Conclusions

Treatment-induced autoimmune diseases may interfere with the effective treatment of HCV. Treatment of such complex patients requires flexible, specific treatment plans and continuous monitoring. This case emphasizes the need for caution in patients with a history of IFN-induced disease, as well as the potential late effects and possible effects of entecavir use in a patient with PEG-IFN-α-induced UCTD disorder. Patients needing rituximab but at risk of HBV reactivation were safely given entecavir, as it was well-tolerated when used in accordance with approved guidelines and recommendations. This is the first case reported as EDAC, a possible delayed complication of PEG-IFN-α plus ribavirin and entecavir.
